# Supplementary material for: Novel and efficient synthesis of insulating gas- heptafluoroisobutyronitrile from hexafluoropropylene
Source: R Soc Open Sci. 2019 Mar 13;6(3):181751. doi: 10.1098/rsos.181751 (PMC6458427; doi:10.1098/rsos.181751)
Supplement: Supporting Information [file rsos181751supp1.pdf]

Supporting Information

Novel and Efficient Synthesis of Insulating Gas- Heptafluoroisobutyronitrile from Hexafluoropropylene

Zhanyang Gao,[a] Min Wang,[a] Shiyao Wang,[a] Yi Wang,[a] Ruichao Peng,[a] Ping Yu,[a] Yunbai Luo*[a]

[a.] Engineering Research Center of Organosilicon Compounds & Materials, Ministry of Education, College of Chemistry and Molecular Sciences, Wuhan University, Wuhan, 430072, P. R. China

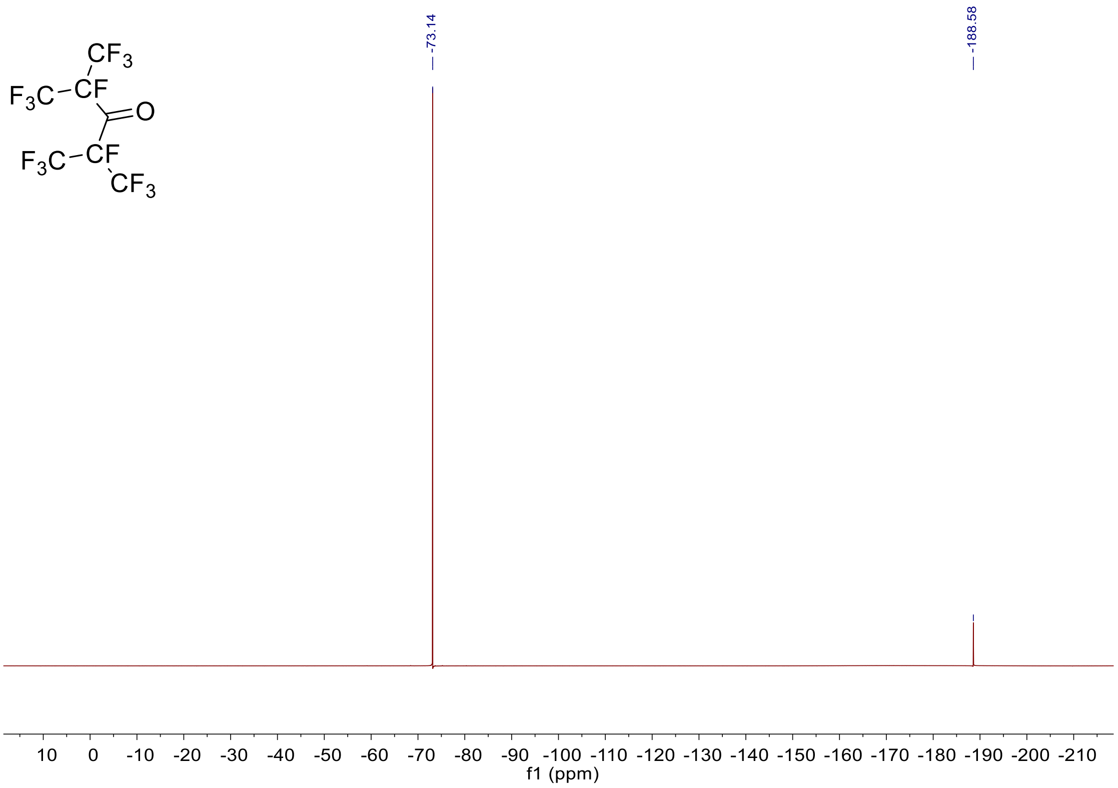

Figure S.1. ^{19}F NMR ($CDCl_3$ 376 MHz) of bis-(perfluoroisopropyl) ketone

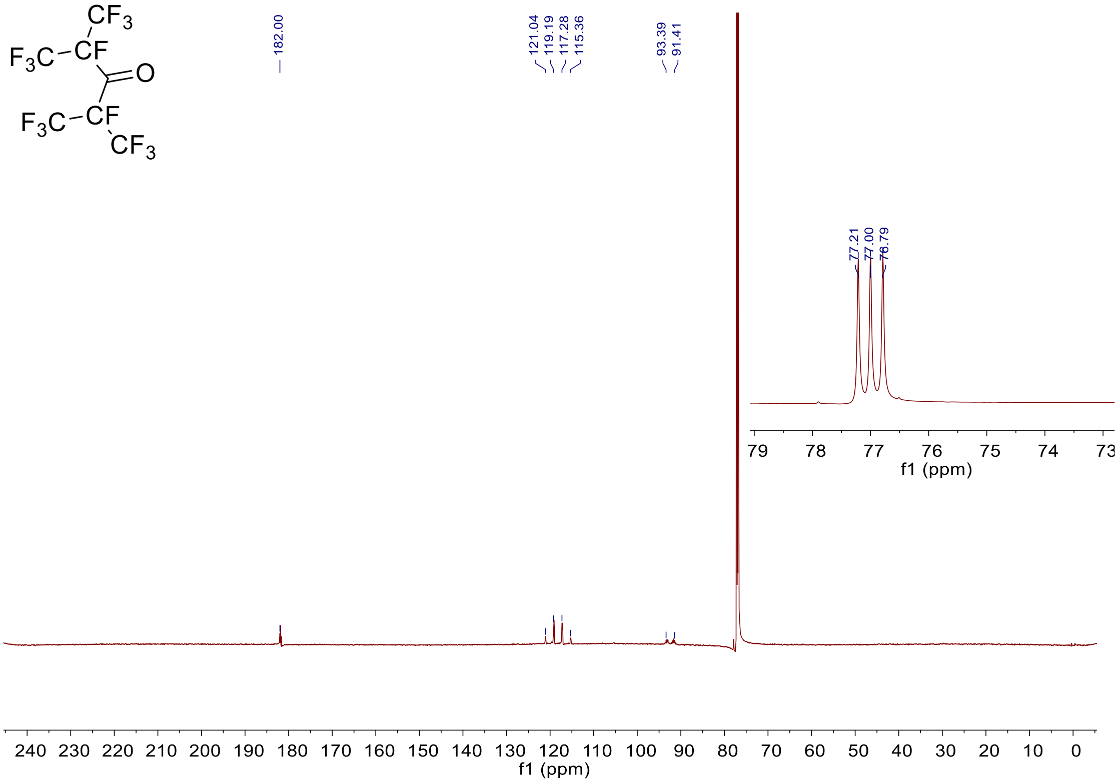

Figure S.2. ^{13}C NMR ($CDCl_3$ 126 MHz) of bis-(perfluoroisopropyl) ketone

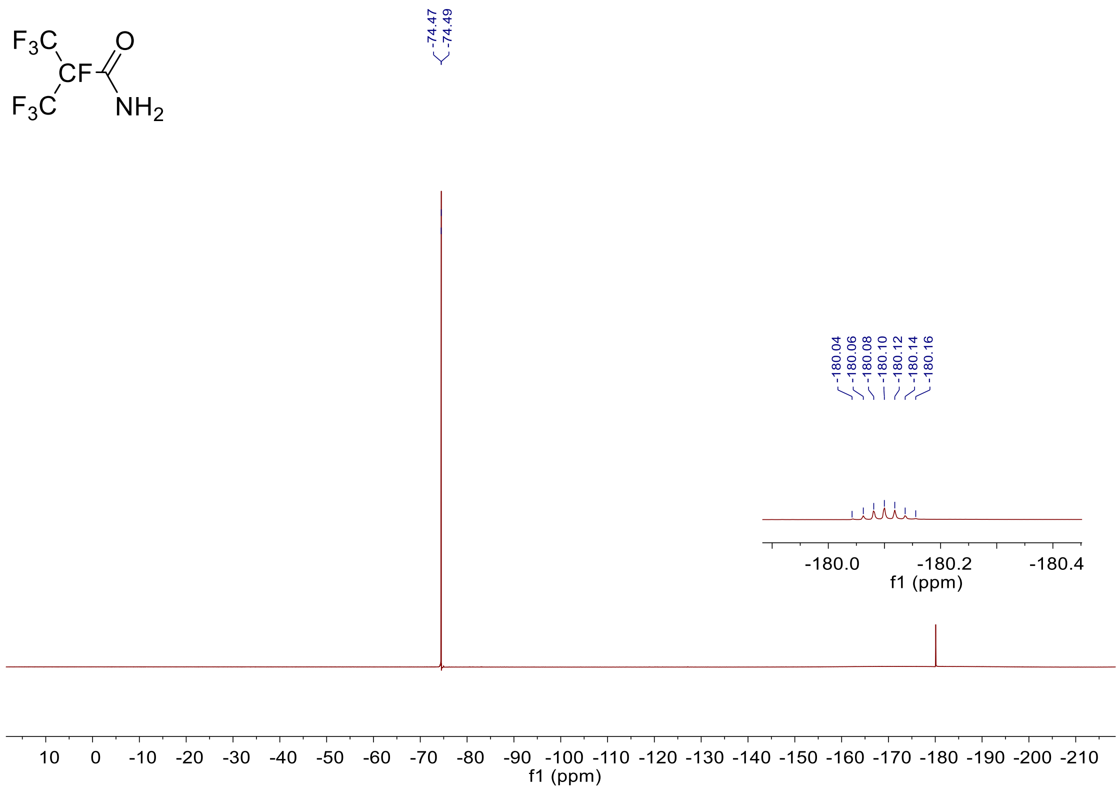

Figure S.3. ^{19}F NMR ($CDCl_3$ 376 MHz) of heptafluoroisobutyramide

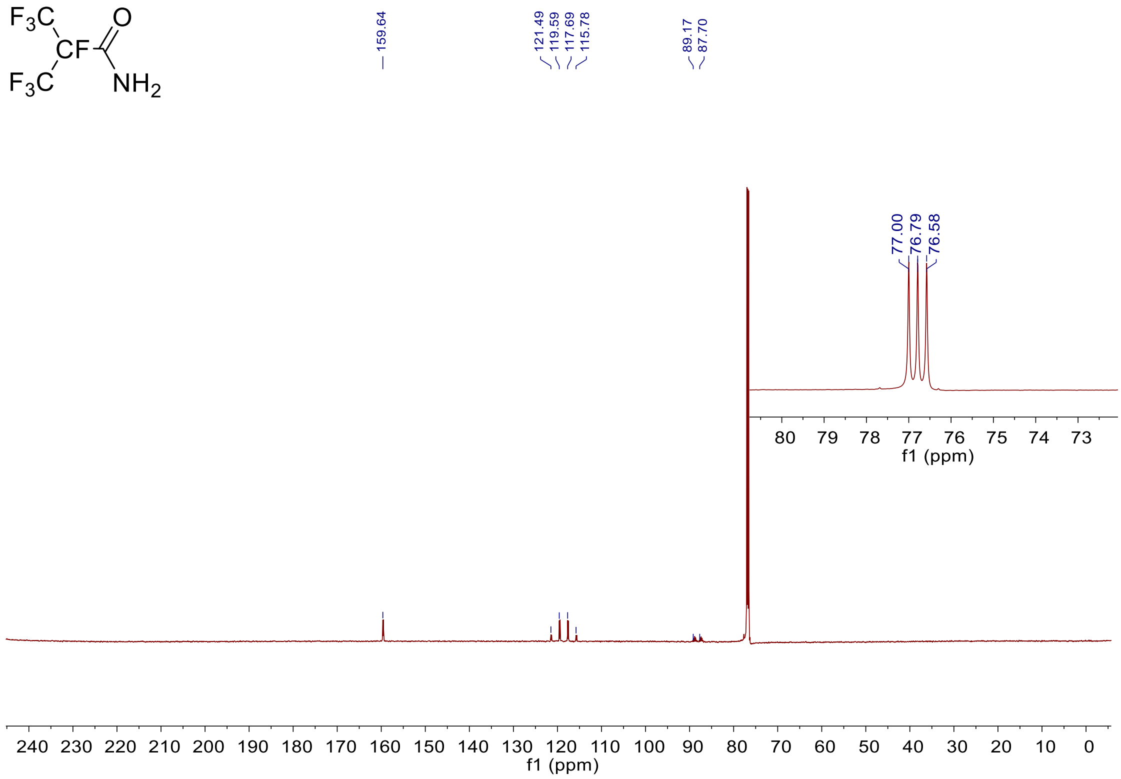

Figure S.4. ^{13}C NMR ($CDCl_3$ 126 MHz) of heptafluoroisobutyramide

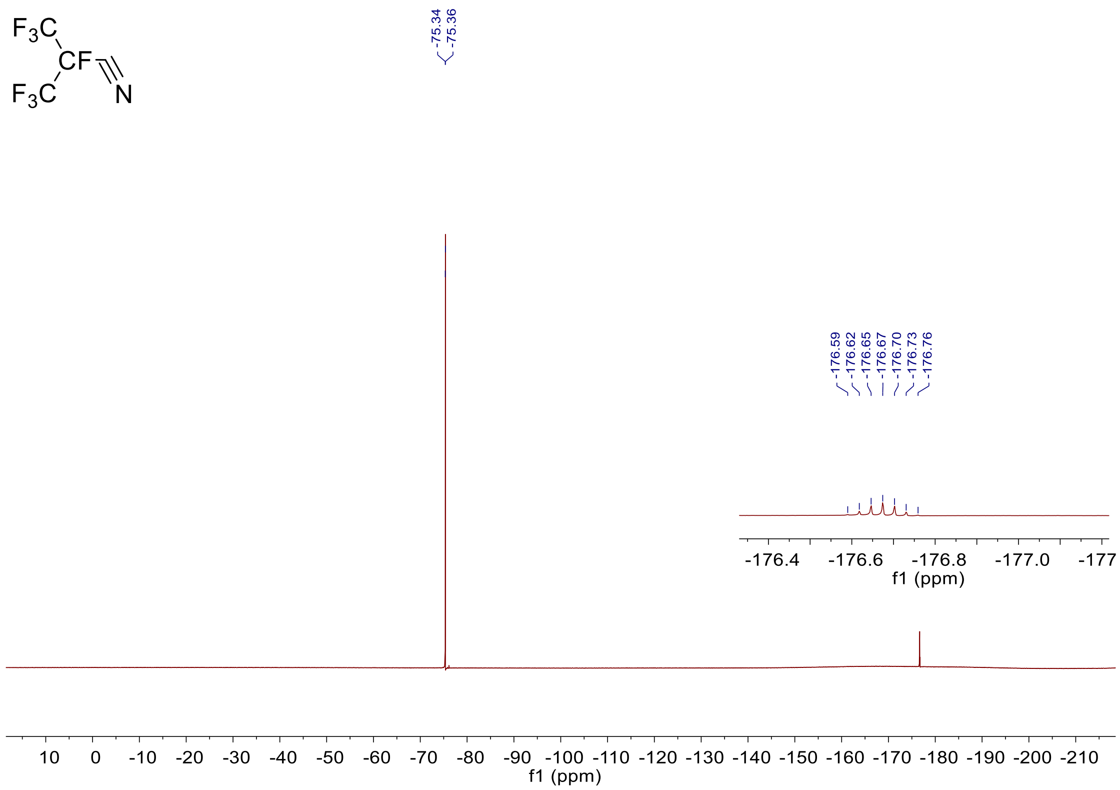

Figure S.5. ^{19}F NMR ($CDCl_3$ 376 MHz) of heptafluoroisobutyronitrile

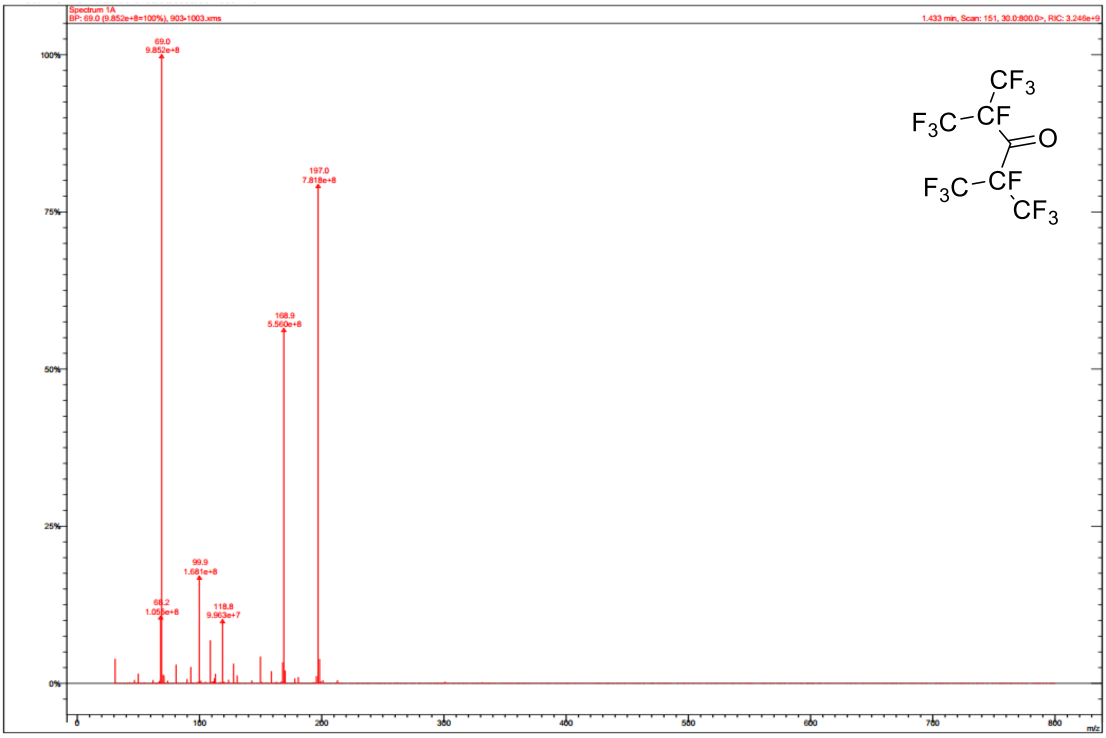

Figure S.6. EI/MS of bis-(perfluoroisopropyl) ketone

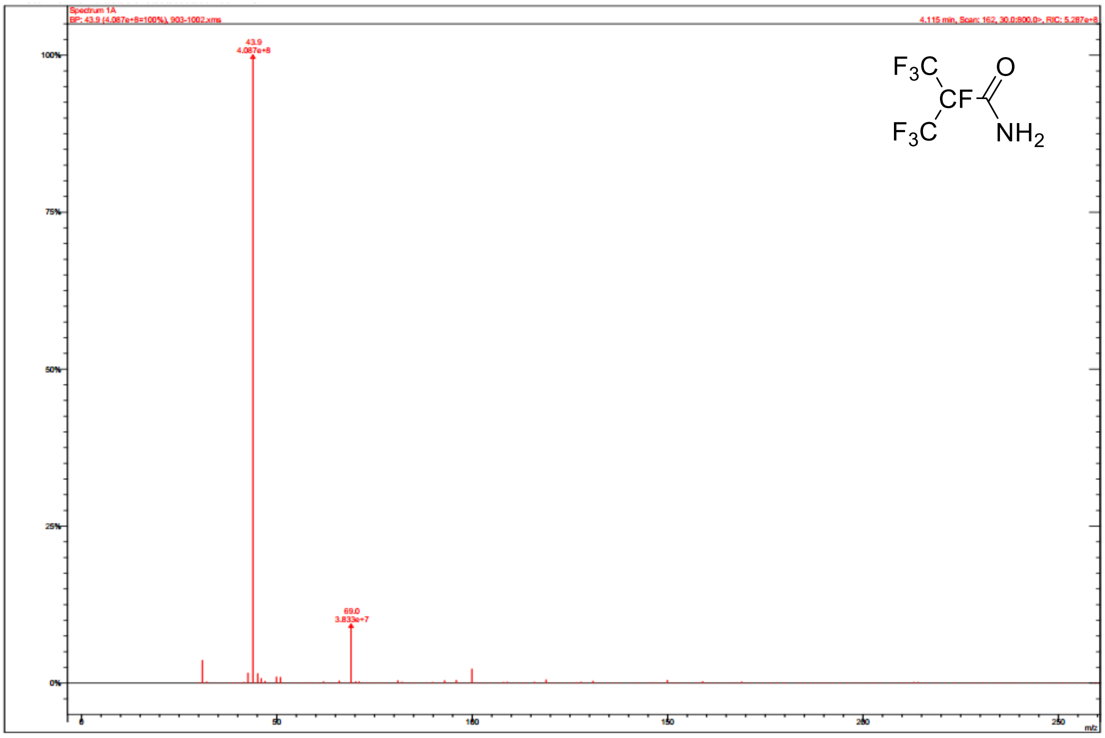

Figure S.7. EI/MS of heptafluoroisobutyramide

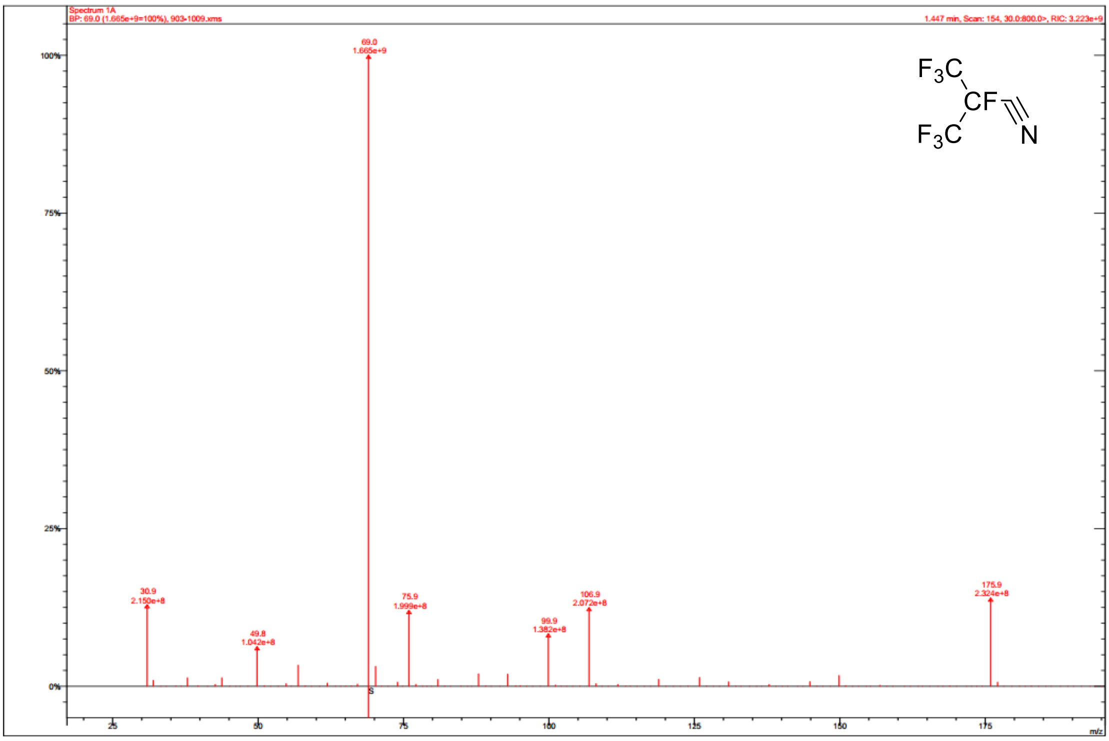

Figure S.8. EI/MS of heptafluoroisobutyronitrile

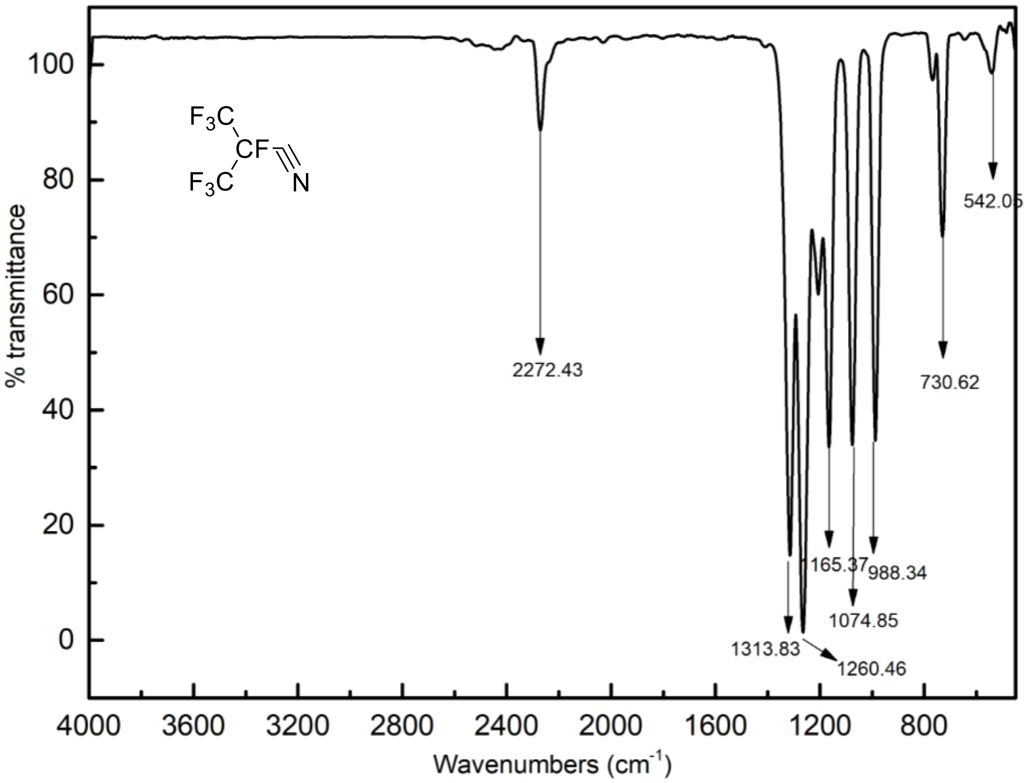

Figure S.9. FT-IR of heptafluoroisobutyronitrile
